# The Epithelial-to-Mesenchymal Transition (EMT) in the Development and Metastasis of Malignant Pleural Mesothelioma

**DOI:** 10.3390/ijms222212216

**Published:** 2021-11-11

**Authors:** Valeria Ramundo, Giada Zanirato, Elisabetta Aldieri

**Affiliations:** 1 Department of Oncology, University of Torino, 10126 Torino, Italy; valeria.ramundo@unito.it (V.R.); giada.zanirato@edu.unito.it (G.Z.); 2 Interdepartmental Center for Studies on Asbestos and Other Toxic Particulates “G. Scansetti”, University of Torino, 10126 Torino, Italy

**Keywords:** epithelial mesenchymal transition, malignant pleural mesothelioma, transforming growth factor β, oxidative stress, miRNAs

## Abstract

Malignant pleural mesothelioma (MPM) is an aggressive tumor mainly associated with asbestos exposure and is characterized by a very difficult pharmacological approach. One of the molecular mechanisms associated with cancer onset and invasiveness is the epithelial-to-mesenchymal transition (EMT), an event induced by different types of inducers, such as transforming growth factor β (TGFβ), the main inducer of EMT, and oxidative stress. MPM development and metastasis have been correlated to EMT; On one hand, EMT mediates the effects exerted by asbestos fibers in the mesothelium, particularly via increased oxidative stress and TGFβ levels evoked by asbestos exposure, thus promoting a malignant phenotype, and on the other hand, MPM acquires invasiveness via the EMT event, as shown by an upregulation of mesenchymal markers or, although indirectly, some miRNAs or non-coding RNAs, all demonstrated to be involved in cancer onset and metastasis. This review aims to better describe how EMT is involved in driving the development and invasiveness of MPM, in an attempt to open new scenarios that are useful in the identification of predictive markers and to improve the pharmacological approach against this aggressive cancer.

## 1. Introduction

Malignant mesothelioma is a rare, aggressive and treatment-resistant tumor that originates from mesothelial cells lining the serous cavity, but the most common type of this tumor is malignant pleural mesothelioma (MPM). MPM development has been closely associated with asbestos exposure [1]; although asbestos use was banned in most countries, the worldwide incidence of MPM continues to climb, so the understanding of asbestos-induced MPM pathogenesis is crucial in order to combat this aggressive cancer. Asbestos fiber effects are considered to be crucial in driving several pathogenetic mechanisms involved in the MPM onset, such as chronic inflammation, oxidative stress and epithelial-to-mesenchymal transition (EMT) [2]. Particularly, the critical role of EMT in tumor progression has been well documented in different types of carcinomas (e.g., breast, pancreas and colon) [3]. Furthermore, EMT and oxidative stress are involved in cancer metabolic changes, and both events are related to the effects mediated by the cytokine transforming growth factor β (TGFβ) [4]. TGFβ is the main inducer of EMT and can promote reactive oxygen species (ROS) production and downregulate the expression of antioxidant enzymes [5]. Moreover, many studies showed that miRNAs and other non-coding RNAs are involved in mesothelioma development and the EMT process [6].

Finally, EMT is among the various processes that influence the MPM tumor microenvironment, thus also providing useful diagnostic biomarkers and therapeutic targets, and a better understanding of EMT can be used to develop a greater number of strategies against this aggressive type of tumor.

## 2. Malignant Pleural Mesothelioma

Malignant pleural mesothelioma (MPM) arises from the pleural surface, and it has been associated with previous (usually occupational) asbestos exposure and is more common in males [7]. The therapeutic approaches against MPM consist of multiple modalities: surgery, chemotherapy and radiotherapy [8]. Despite the efficacy of chemotherapeutic agents used, such as cisplatinum and pemetrexed, with or without bevacizumab, the frequent chemoresistance of MPM to these treatments causes a median survival from diagnosis of only one to two years [8]. Recently, immunotherapy was approved for MPM treatment because of the close relationship between the immune system and mesothelial cells and particularly because of the increasing importance of the role of immune TME in asbestos-induced MPM [9]. Currently, a combination of immune checkpoint inhibitors (ICIs), monoclonal antibodies (mAbs), anti-programmed cell death 1 (PD-1), nivolumab and anti-CTL antigen 4 (CTLA-4) ipilimumab [10] was approved by the Food and Drug Administration (FDA) for first-line treatment of unresectable MPM based on CheckMate 743, which is the first phase of a randomized trial showing evident improvements in terms of survival with immunotherapy in MPM patients [10].

Based on the predominant cell type, MPM is classified into three histological sub-types: epithelioid, sarcomatoid and biphasic. The epithelioid form is more common and related to a better prognosis than the non-epithelial one. Phenotypically, MPM cells are a polygonal or cobblestone-like shape in the epithelioid histotype, while a spindle-like form has been associated with the sarcomatoid MPM type [11]. MPM diagnosis is based on imaging modalities and the evaluation of diagnostic and prognostic biomarkers, such as mesothelin, BRCA-1 associated protein (BAP1), osteo-pontin, fibulin 3 and cytokeratins. Although the role of biomarkers is not well established yet, the latter are used to confirm the MPM histotype through a cytological analysis of pleural effusion and biopsies [7]. Concerning asbestos-related MPM, Klebe et al. interestingly showed an elevated amosite asbestos fiber content using electron microscopy in sarcomatoid MPM samples and highlighted the usefulness of cytokeratin and calretinin immunohistochemistry in discriminating between different MPM histotypes [12]. However, the evaluation of asbestos fiber count by microscopy and mesothelioma biomarkers via immunohistochemistry or blood tests is not effective in confirming mesothelioma diagnosis alone.

In various studies, MPM was associated with tumor-suppressing mutations, such as *BAP1*, neurofibromatosis type 2 (*NF2*), *TP53* and *CDKN2A* [1,13]. Their aberrant expression has been shown to affect MPM cell mobility, proliferation and apoptosis, leading to the onset of the tumor. Finally, MPM has been demonstrated to be triggered by different viral and environmental agents, such as Simian Virus 40 and ionizing irradiation [14], but the main risk factor is still exposure to asbestos fibers.

## 3. Asbestos Effects and MPM Development

The term “asbestos” refers to six types of hydrated mineral silicate fibers in nature classified into serpentine (chrysotile) and amphibole (crocidolite, amosite, anthophyllite, actinolite and tremolite) forms. Asbestos toxicity depends on the dose and length/diameter ratio [15], so amphibole fibers are more dangerous, and their biopersistence is longer than serpentine ones [1].

Asbestos exposure has been related to different diseases, such as asbestosis (a form of pulmonary fibrosis) and MPM, the latter of which is clinically evident after a long latency period of about 40 years [16], and different pathogenetic mechanisms promoted by asbestos exposure were demonstrated to be involved in MPM development. After inhalation, asbestos fibers migrate to the pleura by inducing irritation, damage and fibrosis [1,15,16,17,18]. Moreover, asbestos fibers can induce chronic inflammation, involving TNF-α and IL-1β release by macrophages, and, consequently, macrophagic frustrated phagocytosis [18], mitosis aberrations and the generation of free radicals, the latter of which is involved in promoting DNA, lipid and protein damage. Moreover, asbestos exposure induces the release of different molecules, such as growth factors or cytokines (such as, TGFβ, HGF and VEGF), which are widely involved in asbestos-related pathologies onset. These asbestos-induced mechanisms promote cell necrosis and the release of damage-associated molecular patterns (DAMPs), such as high mobility group box 1 protein (HMGB1) involved in survival, proliferation and autophagy, particularly in mesothelial asbestos-exposed cells [8,19]. Therefore, asbestos fibers were demonstrated to allow, through these events, the creation of a microenvironment prone to the cancer onset and progression. Thus, asbestos fibers can induce epithelial and mesenchymal plasticity; therefore, asbestos-related diseases have been associated with epithelial-to-mesenchymal transition [20,21,22].

## 4. Epithelial-to-Mesenchymal Transition

**Figure 1:**
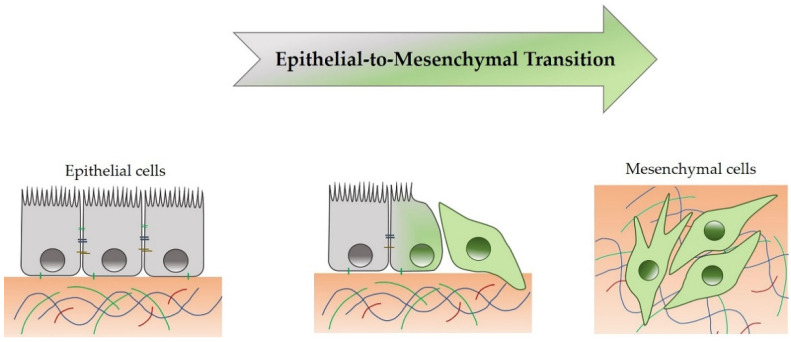
Transition from epithelial cell toward a mesenchymal phenotype.

The epithelial-to-mesenchymal transition (EMT) is a morphogenetic reversible process, during which an epithelial cell loses its own features by reducing some epithelial proteins expression (e.g., E-cadherin, β-catenin, cytokeratins-5/6 and ZO-1) and by acquiring a mesenchymal phenotype, thereby increasing mesenchymal proteins expression (e.g., N-cadherin, Fibronectin, Vimentin, α-SMA, Snail, Slug, TWIST, ZEB-1/2 and MMP-2/9) [3,21,23]. Therefore, epithelial cells lose the apical-basal cell polarity and disassemble intercellular (tight junctions and desmosomes) and cell-ECM junctions (e.g., integrins and fibronectin) [21]. During EMT, a complete mesenchymal state is rarely reached, but an intermediate state between epithelial and mesenchymal status is reached, as shown in Figure 1.

EMT is crucial in physiological events, such as embryogenesis (type I) and wound healing (type II). However, EMT has also been demonstrated to be involved in cancer (type III) and fibrosis development [3]. In tumor progression, EMT makes cancer cells proficient in mobility, invasiveness, apoptosis resistance and extracellular matrix (ECM) production [3,21,23], thus contributing to the tumor onset.

Mesenchymal-to-epithelial transition (MET) is the reverse process of EMT, in which cells with a mesenchymal phenotype acquire an apical-basal cell polarity, reorganize the cytoskeleton and return to an epithelial phenotype [21]. Both EMT and MET occur in physiological and pathological conditions, and their deregulation has been shown to confer to cancer cell aggressiveness, which is particularly crucial for the establishment of metastasis during tumor progression [3].

### 4.1. EMT in MPM Development and Metastasis

**Figure 2:**
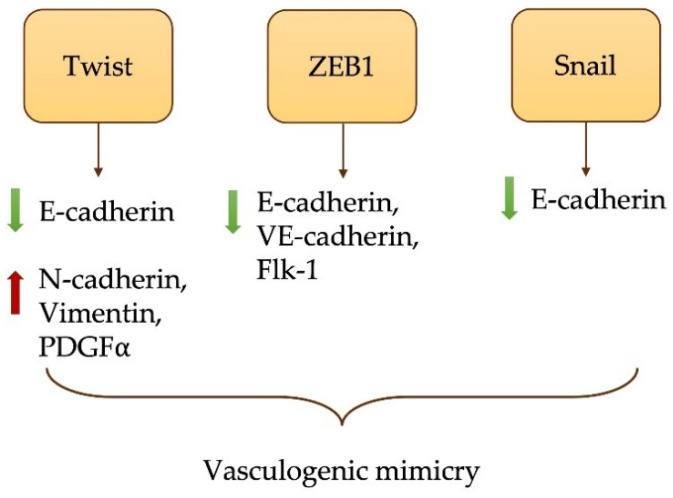
Correlation between EMT induction and VM.

In the different biological contexts previously mentioned, EMT plays a critical role and is regulated by different combinations of EMT-transcription factors (EMT-TFs), including SNAIL-1, Twist and ZEB-1. EMT-TFs exert their effect on gene sequences encoding epithelial or mesenchymal proteins, downregulating the expression of the former and overexpressing the latter [23]. EMT-TFs have been associated with different types of cancer when overexpressed, such as breast cancer, melanoma and hepatocellular carcinoma [24,25,26] and are involved in other functions, besides proliferation and stemness, such as angiogenesis and immunosuppression. In this regard, the crosstalk between EMT and immunosuppression of TME is well established. Tumors need vasculogenic mimicry (VM) to support tumor growth and progression; EMT-TFs can contribute to the VM process [27]. In hypoxic conditions, Twist is overexpressed and targets downstream platelet-derived growth factor α (PDGFα), while ZEB1 supports tumor progression through EMT induction and VM processes. Moreover, Snail upregulation in tumor cells promotes VM event via E-cadherin repression (Figure 2).

Moreover, evidence of VM has been shown in human mesothelioma cell lines alone and those co-cultured with human umbilical vascular endothelial cells, as well as cells immunohistochemical labeled with tumor-associated vasculature of human mesothelioma cells xenotransplanted into mice [28]. This evidence encourages anti-angiogenesis therapy, such as anti-angiogenic tyrosine kinase inhibitors or Bevacizumab (anti-VEGF mAb) [29]. Furthermore, cells of TME, such as cancer-associated fibroblasts (CAFs), tumor-associated macrophages (TAMs), endothelial cells and secreted cytokines, strongly affect EMT, tumor growth and metastasis; Duda et al. showed that CAFs promote lung metastasis through the selective depletion of CAFs in lung metastasis [30] via TGFβ secretion. EMT has been shown to be an important event also involved in MPM development [31], hence the EMT inhibition, as reported by Ramesh et al. [32], could also represent a potential therapeutic approach against MPM via the negative regulation of EMT-TFs.

Several studies have shown that asbestos can induce EMT; human mesothelial cells exposed to asbestos fibers promote the release of HMGB1 and TNF-α, which in turn mediate EMT, thus ensuring cell survival and protecting cells from asbestos cytotoxicity [8]. Furthermore, asbestos fibers can induce the expression of profibrotic genes, thus promoting EMT in A549 lung epithelioma cells through the MAPK/Erk pathway, with a modulation of EMT-related proteins α-SMA and E-cadherin [22]. Moreover, EMT markers’ expression and apoptosis resistance were strictly associated with transglutaminase-2 activity in mesothelioma cancer stem cell spheroids [33], thus confirming the involvement of EMT in MPM development.

The association between mesothelin and EMT is well known; in human mesothelial cells (MeT-5A), mesothelin can downregulate the expression of epithelial EMT genes, such as E-cadherin, and upregulate mesothelial EMT and stemness markers, such as Twist and Snail [34]. Furthermore, in the different MPM histotypes, a differential expression of mesenchymal and epithelial markers was found; in sarcomatoid MPM samples, γ-catenin is usually reduced, while Vimentin and Twist are increased. This combination of EMT markers is associated with a prognostic value [35], so a γ-catenin–Vimentin–Twist combination could be considered an MPM prognostic pattern. A prognostic value has even been associated with EMT markers in malignant mesotheliomas [20] and in metastatic lung adenocarcinomas [36]. To underline the crucial role of Twist in the EMT-associated aggressiveness of the MPM, the association between the presence of Twist in MPM and a poor prognosis was highlighted [37]. Moreover, it has been shown that ZEB-1 and Twist are also overexpressed in mesothelioma and primary lung adenocarcinomas, and in the latter, SNAIL-1 overexpression has been associated with a poor survival rate [36]. The genes regulated by SNAIL and ZEB include matrix metalloproteases (MMPs), which are EMT markers that allow the degradation of the ECM, an essential step in tumor progression and the onset of metastasis [23]. MMPs-2 are particularly upregulated during asbestos-induced EMT in both BEAS-2B lung bronchial cells and in Met-5A [38] mesothelial cells.

EMT can be induced by various agonists and in different cell types, such as MPM cell lines [39]. Among these inducers of EMT, which can be also released by the tumor cell, there are growth factors such as the transforming growth factor β (TGFβ) and the hepatocyte growth factor (HGF), as well as cytokines, which include the tumor necrosis factor α (TNFα) [3,40]. However, TGFβ is recognized as the master promoter of EMT, as it is involved in inflammatory, pro-fibrotic and tumor EMT-related mechanisms.

### 4.2. TGFβ-Induced EMT in MPM

The multifunctional cytokine TGFβ exists in three isoforms (TGFβ1, TGFβ2, TGFβ3) that derive from a 55 kDa pro-peptide processed by furins into an amino-terminal fragment, the latency-associated peptide (LAP), and another acid carboxy-terminal fragment (mature bioactive TGFβ), non-covalently linked. The latent TGFβ is activated by proteases which cleave the LAP [41,42]. Bioactive TGFβ is a dimeric protein that binds TβRI and TβRII cell surface receptors, thus making a heterotetrameric complex. The kinase domain of TβRII phosphorylates TβRI successfully causes the activation and phosphorylation of SMAD2 and SMAD3 downstream effectors [41]. SMAD2 and SMAD3, in turn, form a trimeric complex with SMAD4, which migrates into the nucleus and works as a transcription factor by regulating the expression of genes involved in proliferation, migration, inflammation and angiogenesis [23,43,44,45], predominantly via an EMT event.

TGFβ has a double function in tumorigenesis; it initially acts as a tumor suppressor by inhibiting cell proliferation, downregulating oncogenes, such as c-Myc, and inducing genes involved in cellular proliferation. Then, after acquiring oncogenes and tumor suppressor mutations, TGFβ becomes pro-tumoral by promoting the production of ECM components via tumor-associated fibroblasts (CAFs) [42,44], angiogenesis and EMT induction [44].

**Figure 3:**
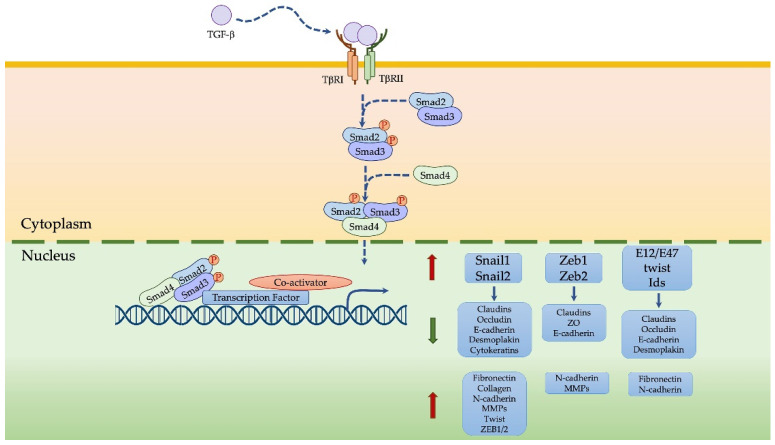
TGFβ activation of EMT transcription factors.

TGFβ induces EMT via SMAD and non-SMAD-dependent signaling pathways [44,46]. In a SMAD-dependent way, TGFβ induces the expression of some transcription factors, including SNAIL-1, Twist and ZEB-1, all involved in EMT and downregulates epithelial markers in particular, such as E-cadherin [23,42,44,47], thus promoting EMT (Figure 3). In a non-SMAD-dependent way, TGFβ-induced EMT involves some signaling pathways, such as mitogen-activated protein kinase (MAPK), PI3K, NF-κB and small GTPases of the Rho family [4,48,49]; among these non-SMAD signaling pathways, PI3K actives Akt, which inhibits GSK-3β in turn, a regulator of SNAI1, SNAI2, Twist and ZEB degradation, thus influencing EMT [4]. Moreover, EMT transcription factors can induce the overexpression of ligands for TGFβ, favoring the EMT program [23]. Finally, TGFβ2 production could in turn be induced by the acidic tumor microenvironment, thus inducing EMT, particularly in mesothelial cells [50]. TGFβ could regulate EMT with post-transcriptional mechanisms as well; during TGFβ-induced EMT, miRNA-200, miRNA-34 and miRNA-205 families are downregulated [43,51], so they cannot negatively exert their effect on EMT-TFs, ZEB1 or SNAIL [52].

TGFβ can be secreted by the immunosuppressive cells of TME: regulatory T cells, TAMs and myeloid-derived suppressor cells (MDSCs) promoting EMT in various cancer cells [53,54]. Woo et al. demonstrated in early-stage non-small cell lung cancer and late-stage ovarian cancer samples increased levels of CD4^+^ CD25^+^ T cells that secreted TGFβ [53], which in turn induce EMT. Furthermore, TGFβ released by TAMs induces VEGF-A expression in mouse macrophages and MMP-9 expression and stemness features in hepatocellular carcinoma, promoting metastasis and invasiveness [55]. Asbestos fibers cause a chronic inflammatory response in mesothelium and hence the release of cytokines, including TGFβ; TGFβ secretion was observed in MPM cell lines, pleural effusions from MPM patients and asbestos-exposed mesothelial cell lines [56,57]. In different studies, TGFβ pathway activation was associated with MPM development [58,59]; it has been demonstrated that the TGFβ-SMAD pathway is activated in malignant mesothelial cells, thus promoting proliferation and ECM production [60]. Furthermore, H2052 and JL-1 mesothelioma cell lines treated with inhibitors of TGFβ receptors reduced invasive growth and MMP2 expression [61]. Moreover, even in H2052 and JL-1 cells treated with pirfenidone, an anti-fibrotic drug, TGFβ-mediated induction of ERK-pathway activation was reduced, suggesting non-canonical TGFβ mediated pathways are also crucial in MPM [62]. Finally, it has been demonstrated that asbestos fibers exposure induces EMT in human mesothelial cells through a mechanism mediated by TGF-β, which in turn, via the SMAD pathway, leads to Twist, ZEB and Snail overexpression [63].

Interestingly, TGFβ may be a potential marker for the diagnosis of MPM from patient pleural effusion, where TGFβ is highly diagnostic and prognostic, particularly in combination with measured mesothelin and IL-10 levels, which increase the sensitivity of the MPM diagnosis and allow stratifying patients in various survival subgroups [64,65,66].

### 4.3. Oxidative Stress-Induced EMT in MPM

Oxidative stress is one of the pathogenetic mechanisms proposed to be involved in asbestos-induced MPM. Previous studies related to EMT and oxidative stress have shown that ROS mediates an oxidative stress status responsible for promoting EMT through a mechanism mediated by GSK-3β, which in turn regulates SNAI1, NF-kB, β-catenin and E-cadherin [3]. It was also demonstrated that hydrogen peroxide (H_2_O_2_) can induce EMT in human mesothelial cells, thus upregulating some stemness genes and TWIST1 in MPM cell lines [67].

Concerning TGFβ and oxidative stress, it has been shown that both can contribute, through crosstalk, to EMT induction [5]; in this context, TGFβ affects ROS production by inhibiting gamma-glutamylcysteine synthetase in lung epithelioma cells [68]. Moreover, hypoxia can promote EMT and an aggressive phenotype in human mesothelial cells through *HIF-1/2α*; HIF-1α regulates the expression of EMT-related genes (TGFβ, Notch, NF-κB, Twist, Snail and Slug), and HIF-2α is involved in the regulation of E-cadherin, Twist and Zeb1 [69]. In hypoxia, EMT is supported by the nuclear translocation of SNAI1 via the inactivation of GSK-3β and by the long-lasting nuclear translocation of β-catenin [3]. Furthermore, heat shock protein 70 is a critical protein that should be involved in oxidative stress and EMT in cancer cell lines (Hsp70) [70]. Hsp70 is a molecular chaperonine that allows survival in stress conditions, and its expression is enhanced in human tumors in response to environmental insults [71,72]. Specifically, Hsp70 inhibits EMT in mesothelial cells through the negative regulation of ROS production, phosphorylation and the nuclear translocation of Smad3 and Smad4 [72]. In addition, VER-155008, an Hsp70 inhibitor in human pleural mesothelioma cell lines (211H, H2452 and H28), suppresses cell growth and enhances autophagy [70]. Therefore, Hsp70 inhibition could be a potential strategy in MPM treatment.

Additionally, it has been described that MPM cells treated with lactic acid and pifithrin-μ, along with HSP70 and p53 inhibitor, underwent necroptosis and apoptosis through oxidative mitochondrial dysfunction and ATP depletion, and moreover induced an EMT-like event [73]. Another protein associated with EMT and oxidative stress is S100A4; it has been demonstrated that this protein is involved in tumor progression and metastasis of different types of cancers, and S100A is found to be important in differentiating sarcomatoid from epithelioid preneoplastic rat mesothelial cell lines in relation to EMT, due to fibronectin and vimentin overexpression, in addition to oxidative stress-related genes overexpression, such as HIF-1α [74].

So, these data show that the EMT-oxidative stress link, particularly in correlation to asbestos exposure, is evident and implicated in MPM development and invasiveness.

### 4.4. MiRNAs as EMT Regulators in MPM Progression

**Figure 4:**
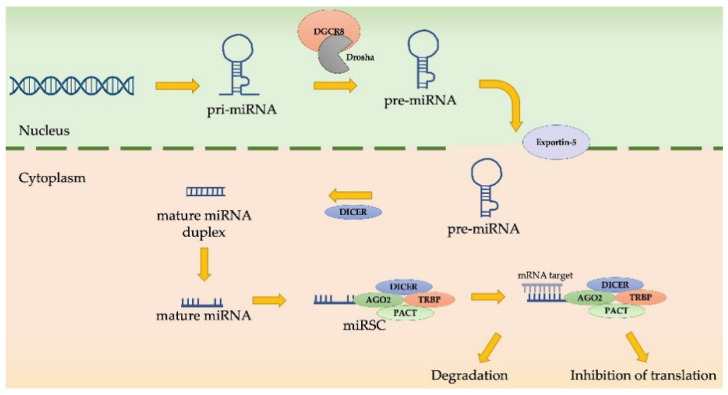
miRNA biogenesis and activity.

MiRNAs are small, non-coding RNAs of about 22 nucleotides in length that are involved in the post-transcriptional regulation of different genes. Nuclear MiRNA biosynthesis begins with a pri-miRNA that is cleaved by a Drosha/DGCR8 complex, thus becoming a pre-miRNA. Then, pre-miRNA is exported into the cytoplasm, is cleaved by Dicer, and binds argonaute proteins to form the miRNA induce silencing complex (miRISC). This complex can be degraded or can inhibit the translation of mRNA targets, based on complementarity with miRNA [75] (Figure 4).

MiRNA expression was altered in various pathological contexts, such as cancer. MiRNA changes are involved in all of the cancer hallmarks, including invasiveness and metastasis, both of which are associated with EMT. Specific EMT-related miRNAs and long non-coding RNAs have been reported to be expressed differently in epithelioid, sarcomatoid and biphasic MPM histotypes [76]; miR-199/214 is overexpressed in sarcomatoid mesothelioma and positively regulated by Twist1 [67], while miR-31-5p is a miRNA highly expressed in the sarcomatoid subtype [77]. MiR-205 is also downregulated in biphasic and sarcomatoid MPM, in correlation to a mesenchymal phenotype and more aggressive behavior [78]. In this context, it has been reported that ursolic acid treatment in MPM cell lines can block EMT by inducing the overexpression of let-7b miRNA, thereby upregulating E-cadherin and downregulating Vimentin [79]. In addition, miR-21-5p targeting programmed cell death protein 4 (PDCD4) involved in apoptosis has been detected in MPM samples and not in normal tissues [80]. This miRNA affects mesothelin expression and could be measured circulating other miRNAs, such as miR-126-3p, miR-625-3p and miR-1031a-3p, and mesothelin to a less invasive diagnosis approach [77,78]. Moreover, miR-145 and miR-205-5p respectively target OCT4 and ZEB-1 and ZEB-2, associated with EMT and tumor invasion capacity [81]. RASSF1C has been observed to downregulate miRNA-33a and modulate EMT markers in lung cancer cell lines [82]. Another EMT-related miRNA is miR-223; in MPM samples it is suppressed, and its target, stathmin, involved in promoting invasion and metastasis, is overexpressed [83].

MiRNAs can regulate TGFβ-induced EMT; the miR-200 family inhibits TGF-β2 mRNA translation and EMT by inducing MET [84]. Moreover, MiR-141 and miR-200c, members of the miR-200 family, are downregulated by TGFβ and EMT inducers ZEB-1 and ZEB-2 [84], and it has been demonstrated that MiRNA-200 increases ZEB-1/-2 [84]. On the contrary, TGFβ can negatively regulate the miRNA-200 family. From a therapeutical point of view, RNA mimics could be exploited to inhibit EMT-associated RNAs via systemic or targeted delivery approaches yet to be defined [75,77].

## 5. EMT and TME Crosstalk in MPM: A Possible Therapeutic Approach

The therapeutic approaches against MPM are surgery, chemotherapy, radiotherapy and recently, immunotherapy [8,9]. Unfortunately, conventional strategies are not always successful. For example, chemotherapy itself has been reported to induce irreversible EMT through endoplasmic reticulum stress, which consequently activates the unfolded protein response both in lung adenocarcinoma cell lines A549 and H358 and primary lung adenocarcinoma samples from patients, providing an interesting mechanism that might contribute to tumor chemoresistance [85].

Therefore, the introduction of new possible therapeutic approaches is necessary. In this sense, the FDA authorization of the ICIs for first-line treatment of unresectable MPM [10] constituted a meaningful improvement in MPM therapy. However, not all MPM patients have a favorable benefit/risk ratio using ICIs, so we need to identify new strategies to treat this type of tumor. Therapeutically, TGFβ-induced signaling pathways could also be potential targets because of their association with induction of the EMT and immunosuppression of TME. In this regard, the inhibition of protein kinase CK-2 by CX-4945 with the use of Ginsenoside 20 (R)-Rg3 in A549 blocked TGFβ-induced EMT [55], along with the use of Galunisertib or Vacosertib (inhibitors of TGFβRI) [86], constituted further potential options in MPM treatment. Moreover, as indicated above, the use of Hsp70 inhibitors or RNA mimics could inhibit EMT and be a potential strategy in MPM treatment [70,75,77].

Interestingly, considering the role of TME, the hypothetical use of mesothelin (MSLN)-targeted CAR T-cells, in combination with checkpoint blockade, could constitute an attractive approach [87]. Recently, Wirawan et al. found that lysine-specific demethylase 1 (LSD1/KDM1), a histone-modifying enzyme, controls mesenchymal phenotype and apoptosis through SNAIL and FAK-AKT-GSK3β [88]. Consequently, the combinatorial therapy of cisplatin and LSD1 inhibitors could be a solution to cisplatin resistance in MPM [88].

Finally, gene therapy could also represent a future strategy by using vectors with a suicide gene or interferons to support the patient’s immune system against tumor cells [89].

## 6. Conclusions

**Figure 5:**
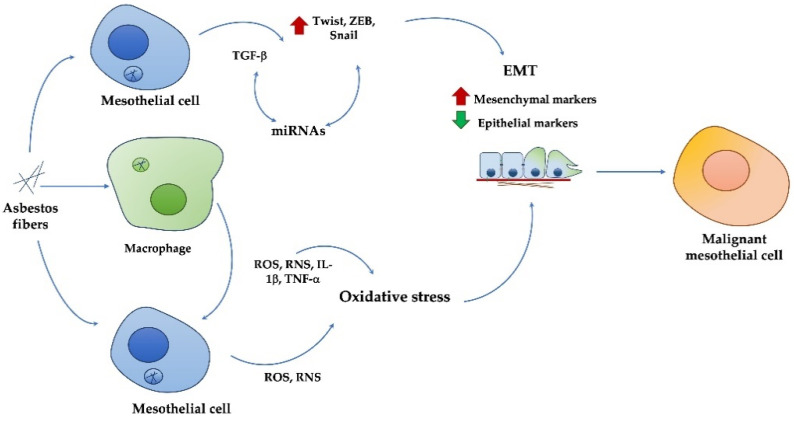
Relationship between EMT, TGFβ, oxidative stress and miRNAs in asbestos-induced MPM development and metastasis.

Malignant pleural mesothelioma (MPM) is such an aggressive tumor that it is currently very difficult to treat. Conventional therapies and current diagnostic biomarkers are not effective. Thus, the identification of new diagnostic markers to identify the early stages of tumor, and the individuation of novel drug targets, to have other available therapeutic approaches, are crucial goals. To reach this aim, it is necessary to understand MPM pathogenetic mechanisms induced by exposure to the main risk factor, particularly asbestos fibers. This review shows how, among events that have been demonstrated to be involved in the MPM onset and progression, EMT is particularly crucial; this process mediates the effects of asbestos fibers on human mesothelial cells through a mechanism mediated by crosstalk between oxidative stress and TGFβ; in addition, miRNAs can cooperate in the MPM development and metastasis via the EMT event (Figure 5).

Taken as a whole, all these mechanisms provide insights that can help improve the diagnosis and treatment of MPM, with the aim of achieving new ways to counteract this aggressive cancer.
